# Editorial: The Interplay Between Epigenetic Regulation and Other Cellular Processes

**DOI:** 10.3389/fgene.2021.691202

**Published:** 2021-05-07

**Authors:** Kai Tang, Huiming Zhang

**Affiliations:** ^1^Department of Horticulture and Landscape Architecture, Purdue University, West Lafayette, IN, United States; ^2^Shanghai Center for Plant Stress Biology, CAS Center for Excellence in Molecular Plant Sciences, Chinese Academy of Sciences, Shanghai, China

**Keywords:** epigenetic regulation, cellular processes, DNA methylation, histone modification, RNA metabolism, lipid, folate, germ cells

Epigenetic changes can influence chromatin structure and, in turn, the accessibility of genetic information as well as the stability of the whole genome. As a result, epigenetic modifications are important to many biological processes, and disruption of epigenetic configuration can lead to developmental abnormalities in plants and mammals, such as failure in tomato fruit ripening (Zhong et al., 2013; Lang et al., 2017) and embryo lethality in mice (Cortázar et al., 2011; Blewitt and Whitelaw, 2013). In addition to coordinating with developmental processes, epigenetic regulation can also play an important role in organisms' responses and adaptation to environmental changes (Etchegaray and Mostoslavsky, 2016; Zhang et al., 2018). Thus, epigenetic processes are tightly regulated in coordination with other cellular processes.

On one hand, cellular processes with important functions can be mediated by epigenetic modifications at the transcriptional level. For instance, Steadman et al. reported that algae cultures treated with 5-aza-2'-deoxycytidine, an inhibitor of DNA methylation, resulted in a remarkable increase in the level of lipid accumulation and increased cell size. Similarly, Zhang M. et al. discovered that DNA methylation regulates fatty acid metabolism and intramuscular fat deposition in chicken. As reviewed in Zhang H. et al., histone deacetylation leads to the initiation and progression of osteoarthritis; while Li et al. showed that knockdown of SETDB1, a histone H3 lysine 9 (H3K9) methyltransferase, resulted in increased levels of reactive oxygen species and impaired proliferation of mouse spermatogonial stem cells.

On the other hand, epigenetic features can be affected by other important biological processes. Certain cellular processes are inherently required for epigenetic modifications, especially DNA methylation and histone post-transcriptional modifications, which are enzymatic processes that involve not only the chromatin but also donor molecules for the modifications. For instance, disruptions in the folate biosynthesis pathway impair the supply of methyl groups for DNA methylation and for histone methylation, resulting in transcriptional desilencing at certain genomic loci in *Arabidopsis thaliana* due to lowered levels of DNA methylation and histone H3K9 dimethylation (Zhang et al., 2012). In this Research Topic, González et al. revealed that, in mouse male germ cells, cocaine caused epigenetic reprogramming of histone modifications involved in gene silencing and the histone-to-protamine replacement, while the effects of cocaine on the histone modifications can be largely blocked by inhibition of the dopamine receptor 1 (DRD1), suggesting a novel connection between the DRD1-dependent dopaminergic system and epigenetic regulation. In *Arabidopsis thaliana*, Zhu et al. observed genome hypomethylation caused by chemical inhibition of the target of rapamycin (TOR), thereby pointing to a connection between epigenetic regulation and this evolutionarily conserved master regulator, which integrates multiple cellular processes to promote growth in all eukaryotes (Dobrenel et al., 2016). As reviewed by Ye et al., the transcription factor ZNF143, which shows higher expression in cancer cells than normal cells, connects promoters to distal regulatory elements and thereby mediates chromatin looping.

Many important biological processes involve RNA metabolism, such as N^6^-methyladenosine that carries many functions in plants as reviewed by Zheng et al., as well as the newly identified miRNAs that silence an important regulator of apoptosis in the report by Coccia et al. Epigenetic regulation of the chromatin are often closely related to RNA metabolism. As reviewed by Zhang J. et al., a crosstalk exists between epigenetic regulation and alternative RNA processing including alternative splicing and alternative polyadenylation. Apparently, the interplay between epigenetic regulation and certain cellular processes can be bidirectional.

The interplay between epigenetic regulation and diverse cellular processes has become increasingly valued over the past few years. While this theme is highlighted by the articles in this Research Topic, the need for a thorough understanding of the epigenetics-connected cellular network continues to urge more discoveries and new insights in this important research area.

## Author Contributions

All authors listed have made a substantial, direct and intellectual contribution to the work, and approved it for publication.

## Conflict of Interest

The authors declare that the research was conducted in the absence of any commercial or financial relationships that could be construed as a potential conflict of interest.
